# Flight altitude dynamics of migrating European nightjars across regions and seasons

**DOI:** 10.1242/jeb.242836

**Published:** 2021-10-25

**Authors:** Gabriel Norevik, Susanne Åkesson, Arne Andersson, Johan Bäckman, Anders Hedenström

**Affiliations:** Department of Biology, Centre for Animal Movement Research, Lund University, Ecology Building, 22362 Lund, Sweden

**Keywords:** Individual-based tracking, Ascent, Descent, Exploratory movement, Wind, Climbing costs

## Abstract

Avian migrants may fly at a range of altitudes, but usually concentrate near strata where a combination of flight conditions is favourable. The aerial environment can have a large impact on the performance of the migrant and is usually highly dynamic, making it beneficial for a bird to regularly check the flight conditions at alternative altitudes. We recorded the migrations between northern Europe and sub-Saharan Africa of European nightjars *Caprimulgus europaeus* to explore their altitudinal space use during spring and autumn flights and to test whether their climbs and descents were performed according to predictions from flight mechanical theory. Spring migration across all regions was associated with more exploratory vertical flights involving major climbs, a higher degree of vertical displacement within flights, and less time spent in level flight, although flight altitude per se was only higher during the Sahara crossing. The nightjars commonly operated at ascent rates below the theoretical maximum, and periods of descent were commonly undertaken by active flight, and rarely by gliding flight, which has been assumed to be a cheaper locomotion mode during descents. The surprisingly frequent shifts in flight altitude further suggest that nightjars can perform vertical displacements at a relatively low cost, which is expected if the birds can allocate potential energy gained during climbs to thrust forward movement during descents. The results should inspire future studies on the potential costs associated with frequent altitude changes and their trade-offs against anticipated flight condition improvements for aerial migrants.

## Abstract (precis)

**Highlighted Article:** Migrating nightjars show vertical displacements at low rates throughout migratory flights, indicating regular adjustments of flight altitude while maintaining flapping flight.

## INTRODUCTION

During active flight, birds use a large fraction of their available power output for forward movement, and behavioural adaptations to reduce the cost of transport are probably selected for (Pennycuick, 1969, 2008). Environmental factors that can have a large effect on the flight budget, such as wind, vary within the air column, suggesting that migrants should respond accordingly; for example, by adjusting flight altitude (Alerstam, 1979; Liechti, 2006; Liechti and Bruderer, 1998; Weber et al., 1998). In level flight, birds usually have a power margin that allows for added demands such as climbing, escape manoeuvres or carrying (fuel) loads (Green and Alerstam, 2000; Hedenström and Alerstam, 1992, 1994). The power margin varies with flight speed, suggesting that a migrant can trade-off energy spent on forward speed against climbing to a specific cruising altitude at the beginning of a flight episode and thereby reduce the total cost of transport by selecting an altitude with favourable tailwind conditions (Hedenström and Alerstam, 1994; Liechti and Bruderer, 1998). The expected fraction of power used (and hence the climb rate) will vary depending on the relative gain (through energy or time savings) of climbing, which is an effect of the relative differences in tailwind conditions (Alerstam, 1985; Hedenström and Alerstam, 1994; Piersma et al., 1997). Although the mechanism of sensing and estimating wind direction and strength at relatively high altitudes is unknown (Hedenström and Åkesson, 2017; Serres et al., 2019), avian migrants are assumed to possess the ability to continuously evaluate the local wind conditions, which would allow them to repeatedly update the current trade-off between forward and vertical flight (Bruderer et al., 1995; Schaub et al., 2004; Senner et al., 2018). By contrast, observations of migrants performing shorter flights during periods of head wind at ground level indicate that birds are unable to predict the weather aloft and need to climb to higher altitudes to evaluate local flight conditions (Liechti, 2006; Schaub et al., 2004). Termed ‘exploratory flights’, such movements are readily distinguished by a steady climb after flight initiation followed by a descent and flight termination. Here, we broaden this definition to also include any larger to-and-fro movements when the bird is airborne at any stage of the migratory flight. Only focusing on the increased power output during climbing may lead to the assumption that extensive vertical movements would elevate the cost of migration and limit the possible benefits of a tailwind at higher altitudes (Galtbalt et al., 2021). But, because of the potential energy gained during ascents, migrants are expected to preserve energy through gliding descents (Baudinette and Schmidt-Nielsen, 1974), which in ideal circumstances could completely balance the climbing costs. That birds switch to a gliding flight in order to recover the potential energy is a common assumption made in models on optimal flight strategies (e.g. Hedenström and Alerstam, 1994; Pennycuick, 1975), which it is now possible to evaluate empirically on an individual level.

In this paper, we present measurements obtained from migrating European nightjars *Caprimulgus europaeus* Linnaeus (henceforth nightjars), a flapping flying bird species that migrates between Eurasia and sub-Saharan Africa (Evens et al., 2017, 2020; Norevik et al., 2017). The overall objectives of the study were to examine the nature and scope of the vertical movements during flights of a long-distance avian migrant, to test predictions from flight mechanical models (see below), and to explore the potential energy costs and benefits associated with vertical displacements during migration. Nightjars are mainly nocturnal birds that migrate during the night and rest motionless throughout daylight hours (Evens et al., 2020; Norevik et al., 2019). As a result, on average 23 flight nights are needed to complete the one-way trip between northern Europe and southern Africa, involving the crossing of the Mediterranean Sea and the Sahara desert, a vast inhospitable area providing limited opportunities for resting and fuelling (Norevik et al., 2017, 2019). The vertical distribution of avian migrants over the Sahara desert broadly follows the trade winds, with a significant shift towards higher altitudes during the spring crossing (Klaassen and Biebach, 2000; Schmaljohann et al., 2009). As migrating nightjars have been observed to fly along considerable detours that may be explained by the energetic benefits of exploiting the winds across the Sahara desert, we wanted to investigate whether the birds do take advantage of the high-altitude trade winds during the spring crossing (Norevik et al., 2020). Under the same assumptions, we expected the occurrence of exploratory flights to vary between seasons and regions depending on the birds’ need to climb to higher altitudes to evaluate local flight conditions (Schaub et al., 2004). Further, our aim was to investigate how often and to what extent nightjars shifted their cruising altitude during migratory flights (for example, rate of ascent and descent), and to examine how these variables varied between seasons and regions. We predicted that the birds’ climb rates would be limited by the power available from flight muscles and that observed maximum rates of climb would be close to the benchmark predicted by aerodynamic theory (Klein Heerenbrink et al., 2015). Furthermore, we wanted to test the assumption that migratory birds generally descend by gliding flight at a sink rate at, or close to, the sink rate associated with the best glide ratio for a bird of nightjar dimensions (Pennycuick, 1969). Finally, we aimed to evaluate the proportion of time spent cruising at level versus altitude shifts during flights as recorded in studies of birds migrating across a rugged landscape (Bishop et al., 2015; Williams et al., 2001).

## MATERIALS AND METHODS

### Deployment and device settings

We deployed 120 custom-made multidata loggers on European nightjars in Sweden (57°N, 16°E) in 2016–2019, with batches of 30 each year. Trapped birds were equipped using a full body harness as described in Norevik et al. (2017). The loggers and harness weigh less than 2.1 g, corresponding to <3% of the body mass of the tagged individual. All animal handling was performed in accordance with approved experimental guidelines and by the Malmö-Lund animal ethics committee (M33-13, M72-15, M74-20 and M470-12). Twenty-six (22%) tags were retrieved until the field season 2020, of which 11 had functioned as expected during a full annual cycle (Table S1). The recapture rate varied between the deployment years with 4 (13%) from 2017 and 12 (40%) from 2016 retrieved. Most tags (21; 81%) were recovered the year after deployment, while 5 (19%) tags were retrieved up to 4 years after deployment. Upon retrieval of tags, we photographed the body and an outstretched right wing of 9 nightjars with a ruler as reference (Pennycuick, 2008). Total wingspan and wing area (including the body between the wings), which were used to parameterise the flight mechanical models used to estimate ascent and descent rates, were determined using ImageJ (v.1.50i) and are provided in Table 1.

**Table 1.**
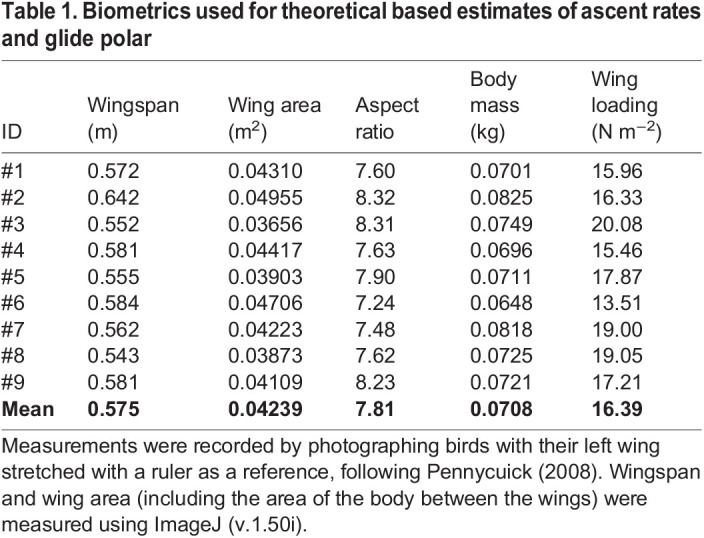
Biometrics used for theoretical based estimates of ascent rates and glide polar

The loggers were programmed to sample flight activity through measurements of acceleration, approximate altitude by air pressure and position by light-level geolocation by light (Ekstrom, 2004), as described in detail in Norevik et al. (2019). The acceleration was sampled in a sequence of measurements along the *z*-axis, approximately parallel to the gravity of a flying bird when mounted on the back. At each sample, a sequence of 5 or 10 measurements (depending on logger model) of 100 ms duration with 5 s between samples was recorded (Fig. S1). This resulted in an activity score between ‘0’ (if no measurement registered activity) and ‘10’ (if all measurements registered activity, indicating active flight). The activity sampling was repeated every 5 min and the distribution of activity categories (0–10 for the pre-2018 loggers or 0–5 for the 2018 and 2019 loggers) was stored every hour. The reason for the shorter sequence of measurements of (and hence a lower distribution of categories for) the 2018 and 2019 loggers was to allocate energy and memory capacity to the 5 min sampling rate of air pressure. We used a Bosch Sensortec BMP280 with temperature compensation and an absolute accuracy of ±1 hPa. The pressure data were converted into altitude above sea level (metres above sea level, masl), using the hypsometric formula (International Organization for Standardization 1975: ISO 2533:1975):
(1)

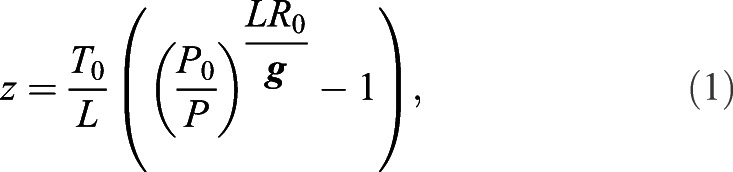
where *T*_0_ is temperature at sea level (assumed to be 288.15 K), *L* is the altitudinal lapse rate of temperature (0.0065 K m^−1^), *P*_0_ is standard atmospheric pressure at sea level (1013.25 hPa), *P* is measured air pressure, ***g*** is acceleration due to gravity (9.8 m s^−1^) and *R*_0_ is the universal gas constant (287.058 J kg^−1^ K^−1^). Following a pre-programmed schedule, sequences of 5 day periods of light-level measurements used for geolocation were distributed over the year (Table S1). Data are available from the Dryad digital repository (http//dx.doi.org/10.5061/dryad.m905qfv1w; Norevik et al., 2021). The light data were primarily used to provide approximate locations of the birds during stationary periods, and the sampling periods were set to overlap the local sunrise and sunset. Measuring light only during selected periods substantially reduced the amount of energy and memory required to process and store continuous light data. Based on previous studies, we assumed that the European nightjars would remain within the longitudinal interval 20°W to 50°E (Norevik et al., 2017), which longitudinally covers the African continent and corresponds to a local time interval of 4 h 40 min. This interval allows us to derive a threshold-based geolocation from the light data (Ekstrom, 2004). For each measurement period, the light intensity was recorded every minute and the maximum value during every 5 min period was stored. The timing of transitions between night and day was extracted using a light threshold level of 2 (light range 0–254) in the software IntiProc v.1.03 (Migrate Technology Ltd), and a sun angle of −6 deg was selected for all devices by matching the derived positions with the breeding area as well as with previously known stationary areas in Europe, the Sahel zone and the wintering area in southern Africa for this population (Norevik et al., 2017). We divided the data into three well-defined migratory episodes: movements within Europe (north of 38°N), the passage of the Mediterranean Sea and the Sahara desert (between 8°N and 38°N), and the movements covering sub-Saharan Africa (south of 8°N).

### Extraction of flight data

We extracted flight segments from the activity data in two steps. First, we assigned hours with high activity level (7 or more registrations of activity category 6 or above, on the 11-grade scale) as core periods of flight (Fig. S2). This usually resulted in periods of several hours with high activity. Second, we added the number of 5 min registrations of activity values of 6 or above in the hour immediately before (pre-core) and after (post-core) the core period to the total duration of flight. Each flight segment thus included a core period of at least 1 h with high activity values and a number of 5 min registrations added at each end of the core period registered during the pre- and post-core hours (Fig. S2). Following Norevik et al. (2019), we extracted flight episodes longer than 3 h, corresponding to a minimum of ∼100 km. This allowed us to filter out potentially non-migratory movements, such as commuting between foraging and roost sites (Evens et al., 2018), whose flight altitude may be under other selective pressures from those of migratory flights. To evaluate the occurrence of exploratory flights, we visually inspected the dataset for shorter flights (<3 h) including a consistent ascent followed by a rapid descent, as indicative for a migrant terminating the flight after sampling the environmental conditions across altitudinal strata (Schaub et al., 2004). For analysis of the possible gliding flight during the final descent, we used the 5 min resolution altitude data of the 2018 loggers to extract time segments of continuous descent following periods of high activity.

### Altitude variation within flights

We quantified the time nightjars spent at near-level flight by extracting the 5 min samples with an absolute vertical difference lower than 30 m from the preceding record (corresponding to an average vertical change of <0.1 m s^−1^ assuming a fixed altitudinal rate of change between samples). This allowed us to quantify the durations of level flight, and to evaluate the relative occurrence of level flight and its seasonal and regional variation. We used two novel approaches to quantify the extent to which the birds alternated their flight altitude. First, we calculated the overall mean vertical change rate of the flights as an indicator of how much vertical motion per unit time is associated with the flights. Second, we calculated the vertical tortuosity of the flight episode as the ratio between the number of shifts between ascent and descent (and vice versa) the birds made during the flight and the number of altitude recordings per flight minus one. This ratio varies between ‘0’ (if the bird made no altitude shifts during a flight, which may occur if it continuously climbs or descends from one level to another) and ‘1’ (if for each altitude registration it continuously shifts between ascending and descending). Given that the initial ascent and the final descent may have a disproportionally large influence in the two approaches, we reran the analyses while excluding these parts of the flights. We tested for seasonal and regional differences in flight altitude variations by using linear mixed models (LMMs) with the described variables as dependent variables and season and region as factorial independent variables, and individual as random intercept. Statistical computations were performed using R v.4.0.4. (http://www.R-project.org/).

### Ascents

From the loggers attached in 2018, we extracted the maximum ascent rate during periods of 5 and 20 min. During active flight, birds can allocate the marginal value between their maximum available power output and the power required for forward flight into climbing (Pennycuick, 1978). The amount of additional power varies between species and may be predicted on the basis of the weight and shape of the birds according to flight mechanical theory (Klein Heerenbrink et al., 2015; Pennycuick, 1969). We estimated the maximum climb rate of the nightjars using the R-package aftp (https://CRAN.R-project.org/package=afpt) based on the average biometrics of our birds: mass 0.0708 kg, wingspan 0.5747 m and wing area 0.04239 m^2^ (Table 1). We derived the maximum ascent rate for three flight speeds: 10 m s^−1^ (*V_z_*=1.16 m s^−1^), which is an observed air speed of red-necked nightjars (*Caprimulgus ruficollis*; Bruderer and Bolt, 2001); 8.65 m s^−1^ (*V_z_*=1.29 m s^−1^), which is the expected maximum speed range of a European nightjar given the biometrics (https://CRAN.R-project.org/package=afpt); and 7.02 m s^−1^, corresponding to the air speed with the maximum rate of climb (1.35 m s^−1^). We compared the observed maximum climb rates with the estimated values using a *t*-test. To evaluate how the maximum climb rates varied with the scope of the total climb during the flight, we fitted a LMM with ascent rate as a dependent variable and total climb as an independent variable, and individual as a random intercept (Bates et al., 2015).

### Descents

All samples of descents during periods of active flight were extracted for analyses of descent rates. To include periods of descent during gliding flight, which is not included in the subset of active flight, we searched the whole dataset of the 2018 loggers for segments of continuous descents after periods of active flight as indicative of a bird descending by gliding. As this procedure occasionally missed parts of the descents occurring after a slight increase in altitude, presumably as a result of a brief period of intermittent flight or an updraft, we chose to run this extraction procedure after smoothing the altitude data with a three-point rolling mean. After excluding the first and last record per descent that may include time when the bird was still in active flight or after it had landed, the descent rate over each period was calculated.

### Exploratory flight events

Wind speed and direction can vary dramatically with altitude, and the airflow at ground level may not allow for a prediction of the airflow at higher altitudes (Liechti, 2006; Liechti and Bruderer, 1998; Schaub et al., 2004; Dokter et al., 2013). Suboptimal flight conditions near the ground could therefore motivate climbs to higher altitudes in search for potential improvements. If the conditions remain poor, the bird is expected to interrupt migration, resulting in a short exploratory flight showing a rather distinct altitude profile consisting of an ascent directly followed by a descent. We scanned the dataset, including flights shorter than 3 h, for flight episodes with an ascent just after the flight initiation followed by a descent and a flight termination where the ascent or the descent resulted in a vertical displacement of at least 500 m and an average vertical speed of more than 0.1 m s^−1^. Given that birds may also check a range of altitudes for flight conditions during cruising flight, we searched for two consecutive vertical displacements of a minimum of 500 m (i.e. an ascent followed by a descent or vice versa) within longer flight episodes.

## RESULTS

**Fig. 1.**
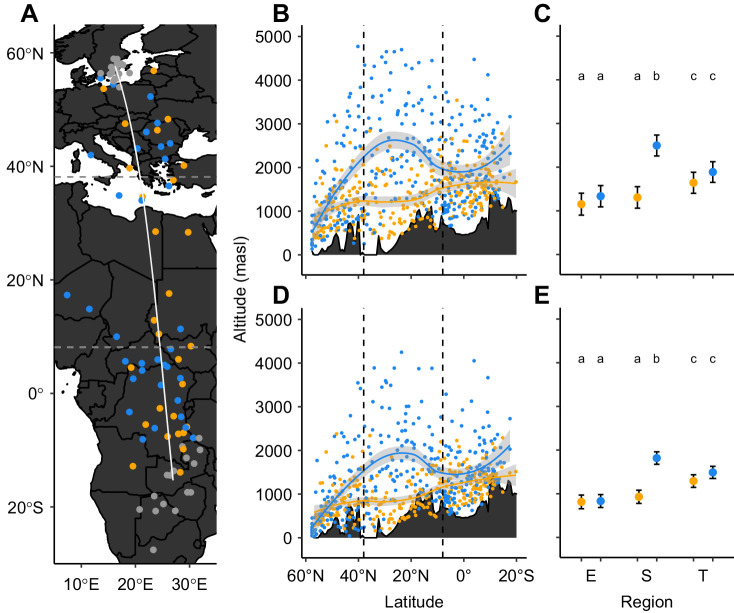
**Autumn (yellow) and spring (blue) altitude distribution of 11 nightjars migrating across Europe, the Sahara desert and sub-Saharan Africa.** Dashed lines at latitudes 8°N and 38°N mark the extent of each region. (A) Circles represent the median positions of sampled geolocation data during breeding and wintering (grey areas), and migration. The curved line represents the great circle track between the breeding site and the median wintering location and was used to sample the ground-level data in B and D (grey contours). The map is in a WGS84 geographic coordinate system. (B,D) Maximum (B) and median (D) daily flight altitude (masl, metres above sea level) for the tracked birds across latitudes. Solid lines are local polynomial fits and the shaded area corresponds to the 95% confidence interval. The landscape reliefs of B and D indicate the ground level along the migration axis, but local variations in ground level will result in flight altitude recordings below this altitude reference. (C,E) Means and their 95% confidence intervals from general linear models for the maximum (C) and median (E) daily flight altitudes for each region (E, Europe; S, Sahara desert; T, sub-Saharan Africa) and season. Different lowercase letters indicate significant differences among means as assessed by *post hoc* Tukey HSD (*P*<0.001).

The tracked nightjars undertook on average 57.1±4.9 (mean±s.d; range 52–69) flight episodes during the roundtrip migrations to and from the wintering range in sub-Saharan Africa (Fig. 1A). Significantly more flights were undertaken in spring (30.2±3.8, range 26–40) than in autumn (26.9±2.9, range 22–31, paired *t*-test=2.39, d.f.=10, *P*=0.034).

### Regional and seasonal flight altitude distribution

Both maximum and median altitude per flight episode varied across seasons and regions (Fig. 1B–E), with highest model means corresponding to the spring crossing of the Sahara desert. Lowest median and maximum flight altitudes were recorded during the spring and autumn flights over Europe along with the autumn flights over the Sahara desert, while autumn and spring flights within sub-Saharan Africa were at significantly higher altitudes. However, referring to the altitude above ground level, the spring flights over the Sahara desert remained the highest flights, while no significant difference was detected between the other categories.

### Altitude variation within flights

**Fig. 2.**
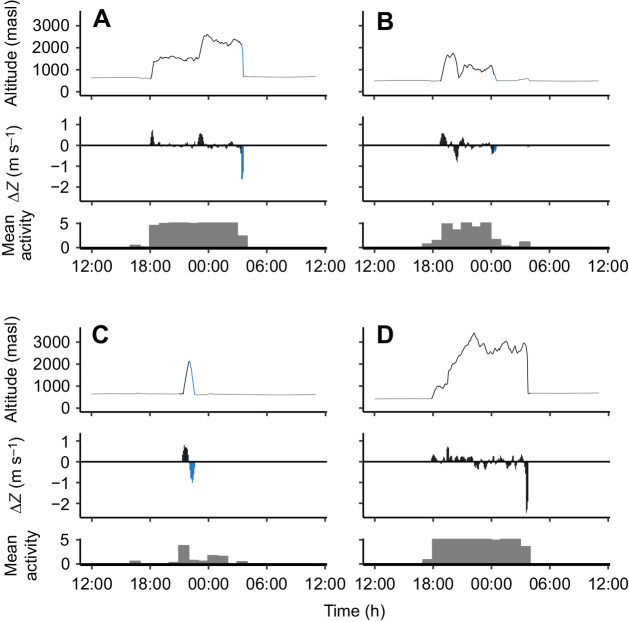
**Four examples of the temporal altitude profile, vertical displacement and flight activity of migrating nightjars.** Upper subplots: the line illustrates the altitude corresponding to the ambient pressure sampled every 5 min. Middle subplots: vertical displacement (Δ*Z*) data are based on a three-point sample rolling mean of the 5 min altitude measurements. Colours of lines and bars represent low or no registered activity (dark grey), continuous flapping flight (black) and periods of low activity while descending, indicating gliding (or intermittent flapping) flights (blue). Lower subplots: bars correspond to the mean activity level (hourly averages) ranging between ‘0’ (no activity registered) and ‘5’ (activity registered in all samples). (A) This flight episode consisted of a relatively rapid climb to ∼1500 m where the bird remained for about half the night before making an additional climb to over 2500 m, before descending by gliding flight to ground level at ∼700 m (date: 11 February). (B) Following an initial climb to ∼2000 m, the bird spent about an hour in near-level flight before undertaking an apparent mid-flight exploratory movement by descending ∼700 m and thereafter ascending to 1200 m, where it remained for the rest of the flight (date: 29 April). (C) After a relatively rapid climb to 2000 m, the bird descended by gliding and landed after 1.5 h flight, illustrating an example of a terminated exploratory movement (date: 23 March). (D) After a relatively slow and irregular ascent to almost 3500 m over 4 h, the bird descended and continued flying for 6 h while alternating between 2500 and 3000 m, before a terminal rapid descent during active flight (date: 14 April).

**Fig. 3.**
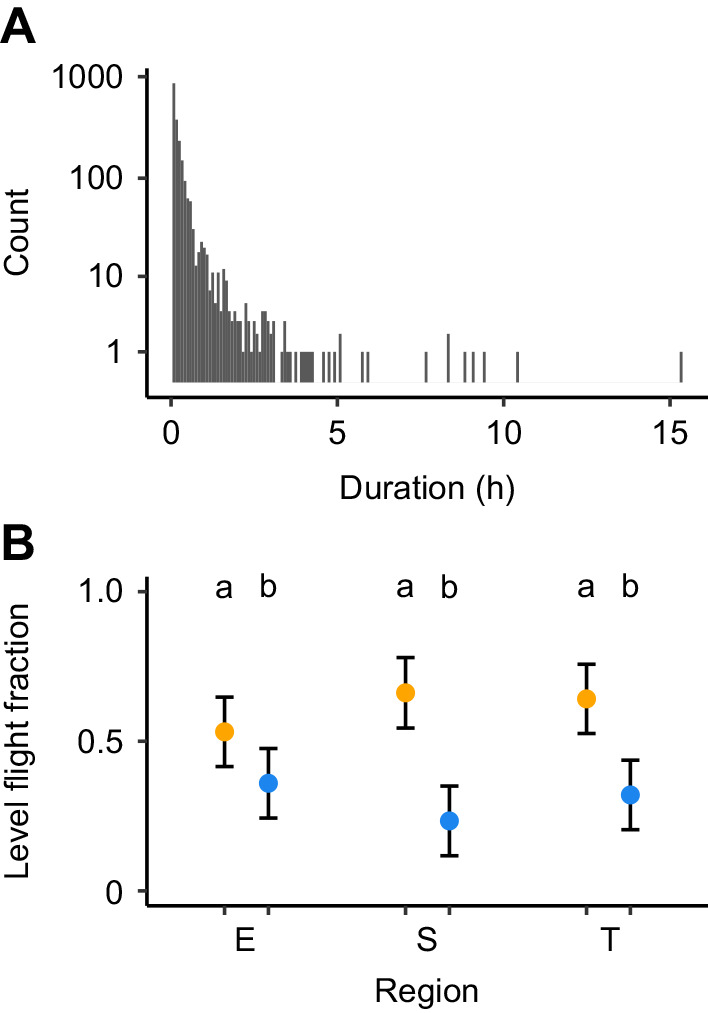
**Distribution of durations of continuous near-level flight and seasonal and regional differences in autumn (yellow) and spring (blue) between Europe, the Sahara desert and sub-Saharan Africa in the fraction of level flight per flight episode.** (A) The distribution illustrates the variation of duration of near-level flight measured as the consecutive number of altitude registrations with less than 30 m difference (corresponding to an average change of less than 0.1 m s^−1^, during each 5 min period between registrations). Bin width corresponds to 5 min and the *y*-axis is a log_10_ scale. (B) Circles and error bars represent means and their 95% confidence intervals from a linear mixed model. Different lowercase letters indicate significant differences among means as assessed by *post hoc* Tukey HSD (*P*<0.05). The level flight fraction is the duration of near-level flight divided by the total duration per flight episode. A value of ‘0.5’ means that half of the flight time was spent in larger vertical displacements than 0.1 m s^−1^, and a value of ‘1’ corresponds to flight without any climbs or descents with a vertical speed above 0.1 m s^−1^.

All birds showed variation in flight altitude within and between flight episodes (Figs 1 and 2), although many changes were generally driven by relatively slow continuous ascent or descent. As a result, vertical displacements of 300 m or more per 5 min segments (corresponding to an average vertical speed of at least 1 m s^−1^) occurred on 296 (1.3%) of the 22,180, 5 min data points during migratory flights. Intermediate altitude changes of 30–300 m occurred on 8725 occasions (39.4%), while near-level flight with changes less than 30 m (on average less than 0.1 m s^−1^) occurred on 13,159 occasions (59.3%). When examining the subset of near-level flight, we note that such flights were typically of short duration and were regularly interrupted by periods of relatively larger altitude changes (Fig. 3A). On a few occasions, near-level flights occurred for more than 3 h (Fig. 3A). The fraction of near-level flight per flight episode were consistently larger during autumn than in spring but did not differ between regions within seasons (Fig. 3B).

**Fig. 4.**
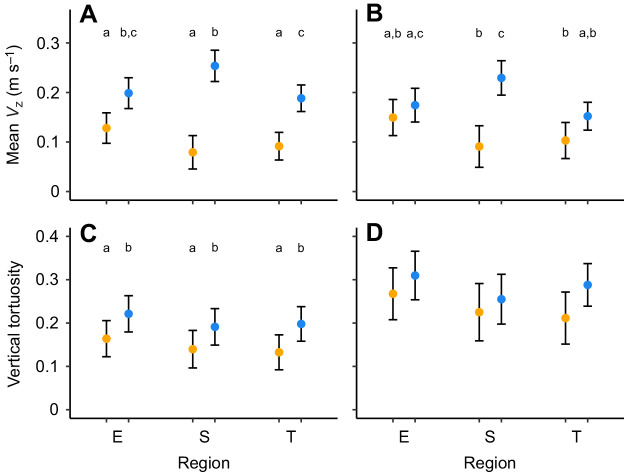
**Seasonal and regional differences in altitude variation during migratory flights of nightjars in autumn (yellow) and spring (blue) between Europe, the Sahara desert and sub-Saharan Africa.** Data correspond to a full flight episode (A,C) and the presumed cruising phase (B,D). (A,B) Mean vertical speed (*V_z_*) per flight episode. (C,D) Altitude change rate, measured as the number of shifts between ascent and descent divided by the total number of altitude registrations per flight episode. Dots and error bars represent means and their 95% confidence intervals from general linear models. Different lowercase letters indicate significant differences among means as assessed by *post hoc* Tukey HSD (*P*<0.05).

The mean (±s.d.) vertical speed for the full flight episode was 0.16±0.10 m s^−1^ (range 0.02–0.54 m s^−1^). As the initial ascent and the final descent may have a large influence on the average vertical displacement of a flight episode, we excluded the first and last segments of vertical displacements to calculate the average speed for the presumed cruising phase (0.15±0.10 m s^−1^, range 0.01–0.53 m s^−1^), which was on average 0.02 m s^−1^ lower than that for the full episode (paired *t*-test: 3.138, d.f.=178, *P*=0.002). The mean vertical speed per flight episode also differed significantly between regions and seasons (Fig. 4A,B). Spring flights were associated with higher average vertical speeds relative to autumn flights, and seasonal differences over the Sahara desert were notable with average vertical speeds in spring more than double those in autumn (Fig. 4A,B).

We also calculated the vertical tortuosity of the flight episodes as the ratio of 5 min recordings that indicated a change from ascent to descent or vice versa divided by the total number of possible changes based on the altitude sampling rate (Fig. 4C; see Materials and Methods). We repeated the analysis after excluding the first climb and the last descent as flights at higher altitudes (e.g. spring flights over the Sahara desert) may involve longer initial ascents and final descents, which will reduce the fraction of tortuosity for the level flight. The mean (±s.d.) rate of change was 0.17±0.08 (range 0.02–0.40) for the complete flight, which was significantly lower than the vertical tortuosity of the presumed cruising phase (0.27±0.13, range 0.04–0.80; paired *t*-test: −9.769, d.f.=174, *P*<0.001). When examining the variation in vertical tortuosity between seasons and regions, the spring flights were more tortuous than the autumn flights within and across regions for the full flight episode, but the significant differences disappeared when only including the cruising phase (Fig. 4C,D).

### Exploratory movements

**Fig. 5.**
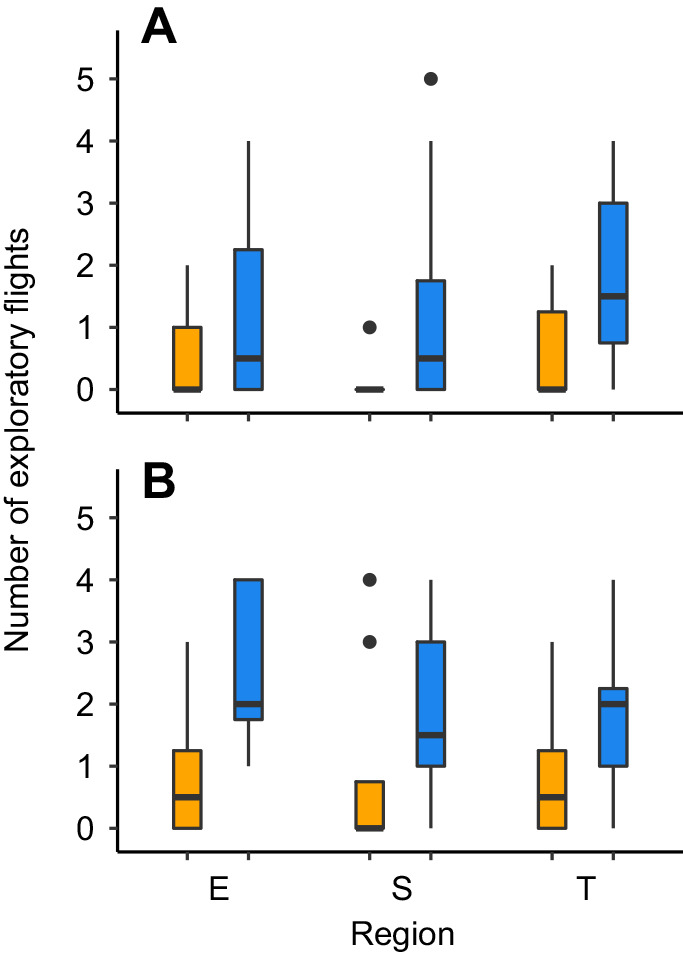
**Seasonal and regional distributions of exploratory movements per migrating nightjar in autumn (yellow) and spring (blue) in Europe, the Sahara desert and sub-Saharan Africa.** (A) Number of exploratory movements that cover a complete (<3 h) flight episode followed by a flight termination (cf. Fig. 2C). (B) Number of flight episodes that include an exploratory movement mid-flight (cf. Fig. 2B). Boxplots show the distribution of flight occurrences [line, median value; box, interquartile range (IQR); whiskers, 1.5×IQR; points, outliers].

We evaluated the occurrence of terminated exploratory movements by scanning the dataset for flights consisting of a climb of a minimum of 500 m followed by a descent with an overall average rate of vertical displacement of more than 0.1 m s^−1^, after which the flight was terminated. This resulted in 45 occurrences of terminated exploratory movements among 8 of 11 individuals where data on location, activity and altitude were available across the annual cycle. We also screened the data of flight episodes using the same settings to identify the occurrence of potential exploratory movements within migratory flights, resulting in another 71 occasions. Exploratory movements were more common in spring than in autumn for both terminated exploratory movements (Fig. 5A; β=1.0417, s.e.=0.3656, d.f.=46, *t*=2.849, *P*=0.00654) and mid-flight exploratory movements (Fig. 5B; β=1.2083, s.e.=0.3610, d.f.=39, *t*=3.347, *P*=0.00182), but there were no significant differences between regions in either mid-flight or terminated exploratory movements. The altitude profile of a mid-flight exploratory movement was not limited to an ascent followed by a descent as in the traditional view of a terminated exploratory movement. A subset of 10 mid-flight exploratory movements started with a descent from a relatively high altitude followed by an ascent.

### Ascent

**Fig. 6.**
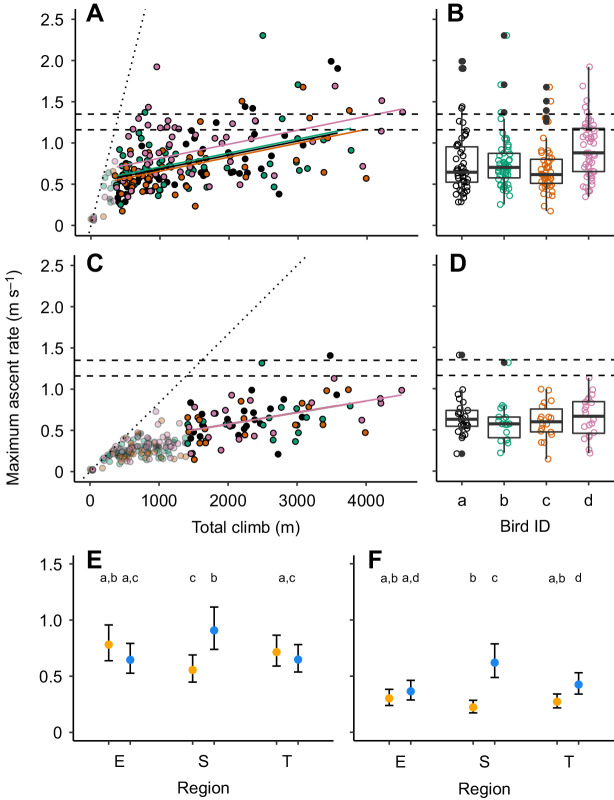
**Maximum ascent rate (vertical speed) per flight episode across the annual cycle.** Data are based on 5 min (A,B) and 20 min (C,D) segments in relation to total climb (A,C) for each bird (B,D), and their seasonal and regional comparisons (E,F). Colours in A–D represent bird ID and dashed horizontal lines correspond to theoretical upper limits of sustainable climb rate of a bird with morphology representing a European nightjar (Table 1) flying with an air speed of 7 m s^−1^ (1.35 m s^−1^) or 10 m s^−1^ (black, 1.16 m s^−1^). The dotted line (A,C) illustrates the maximum climb rates possible given the quantified range of climbs and the resolution of the time segments. For the statistical analyses, we used data where the total ascent per flight episode was larger than the altitude covered by a bird performing a maximum climb rate (1.16 m s^−1^) during the time segment (A: 5 min, C: 20 min) to minimise potential sampling effects. Boxplots in B and D show the distribution of maximum climb rates (line, median value; box, IQR; whiskers,1.5×IQR; solid circles, outliers). E and F show back-transformed model means and their 95% confidence intervals from general linear models for the 5 min (E) and 20 min (F) segments, respectively, of maximum ascent rates per flight episode for each region and season (see Fig. 1). Different lowercase letters indicate significant differences among means as assessed by *post hoc* Tukey HSD (*P*<0.05).

We quantified the maximum ascent rate per flight episode over shorter [5 min, median 0.67 m s^−1^, interquartile range (IQR) 0.42 m s^−1^, full range 0.06–2.30 m s^−1^; Fig. 6B] and longer (20 min, 0.61 m s^−1^, IQR 0.31 m s^−1^, full range 0.15–1.41 m s^−1^; Fig. 6D) periods. These measurements were significantly lower than the theoretical maximum climb rate at 1.16 m s^−1^ of a nightjar-sized bird flying at an air speed of 10 m s^−1^ (Fig. 6A–D; 5 min: *t*-test: −14.52, d.f.=197, *P*<0.001; 20 min: *t*-test: −20.20, d.f.=80, *P*<0.001). In 30 (15%) of the flights, the birds reached vertical speeds above 1.16 m s^−1^ when measured over 5 min, while there were only two registrations with vertical speeds above 1.16 m s^−1^ for the 20 min intervals. The maximum climb rate per flight episode increased with larger vertical displacements for both the 5 min and 20 min recordings (5 min: β=0.000170, *t*=7.527, *P*<0.001; 20 min: β=0.000141, *t*=4.874, *P*<0.001; Fig. 6A,C). This means that the average of maximum climb rates over 5 min was about 50% of the theoretical maximum during flights involving climbs of less than 1000 m, while it approached 100% when associated with climbs of 4000 m. The highest maximum climb rates were achieved during the spring crossing over the Sahara desert (Fig. 6E,F).

### Descent

**Fig. 7.**
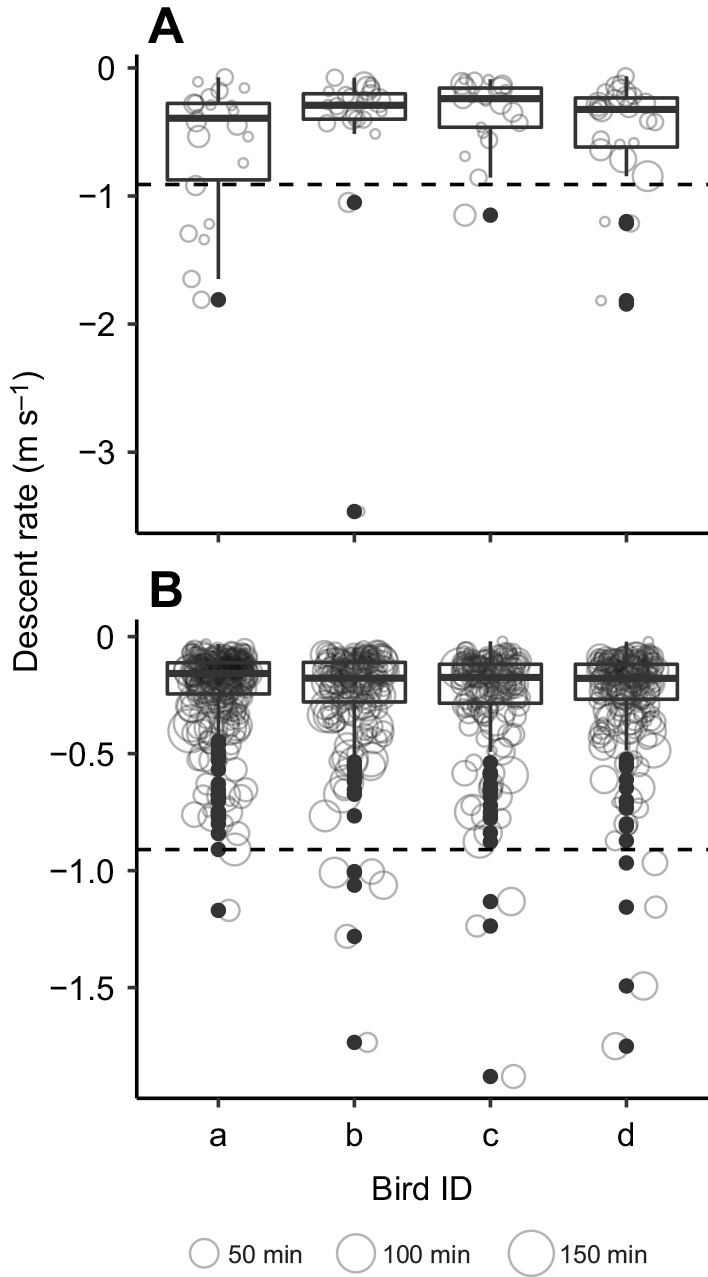
**Vertical change rate during episodes of gliding and active descents of four individuals during migratory flights.** (A) Gliding. (B) Active descents. The size of circles corresponds to the duration of descents (in minutes); distributions of vertical speeds are illustrated with boxplots (line, median value; box, IQR; whiskers, 1.5×IQR; points, outliers). Dashed horizontal lines at −0.91 m s^−1^ represent the descent rate corresponding to a forward speed of 10 m s^−1^ by a nightjar achieving a lift to drag ratio of 11 (Table S2, Fig. S3).

The dataset of descents with a minimal duration of 10 min includes 959 records. The majority (865) of the descents were recorded during periods of active flight, while 94 registrations occurred during periods of low to intermittent flight activity (henceforth referred to as glide). The median vertical change rate during glide was −0.30 m s^−1^ (IQR 0.33 m s^−1^, full range −0.1 to −3.5 m s^−1^; Fig. 7A), while during active flight it was −0.17 m s^−1^ (IQR 0.16 m s^−1^, full range 0.0 to −1.9 m s^−1^; Fig. 7B).

## DISCUSSION

### Regional and seasonal flight altitude distributions

The tracked nightjars reached altitudes just below 5000 masl, which is similar to recordings of another trans-Saharan migrant, the Eurasian hoopoe, *Upupa epops*, but considerably lower than the maximum flight altitudes above 6000 masl of great reed warblers, *Acrocephalus arundiaceus* (Liechti et al., 2018; Sjöberg et al., 2021), and great snipes, *Gallinago media* (above 8000 masl; Lindström et al., 2021). The nightjars flew at the highest altitudes during the spring crossing of the Sahara desert, which conforms well with the general pattern of altitude distribution of avian migrants based on radar observations in Europe and west-Saharan Africa (reviewed in Bruderer et al., 2018). Our data show that the pattern of flight altitudes between seasons recorded at a population level are also present at an individual level. Although formal analyses of wind conditions along the actual tracks of desert-crossing nightjars are objectives of future studies, the seasonal flight altitude pattern of the nightjars suggests that they fly at altitudes where the general airflow is expected to be relatively supportive (Liechti and Schmaljohann, 2007; Schmaljohann et al., 2009).

### Rates of ascent and descent

The mean maximum ascent speeds of nightjars were significantly lower than the maximum climb rates predicted from flight mechanics (Klein Heerenbrink et al., 2015). The finding that the birds for the majority of the time climb at even lower rates indicates that nightjars were rarely operating near their maximum power available during climbs. In fact, the highest climb rates were associated with the high-altitude flights during the spring crossing of the Sahara. Birds generally have large fuel stores before initiating these flights, and tracking studies have revealed that nightjars regularly undertake stopovers of several weeks just south of the Sahara, most likely fuelling before the barrier crossing (Evens et al., 2017; Norevik et al., 2017, 2019). As large fuel loads impair the birds’ capacity to climb (Hedenström and Alerstam, 1992; Pennycuick, 1969), we expect that nightjars would have the least power available for climbs during this period. We note that climbs recorded during 5 min intervals occasionally reach rates far above the predicted maximum climb rates, but that such observations disappear when analysing ascents over 20 min. The occurrence of such apparent elevated bursts of climbs indicates that ascending nightjars may occasionally gain support by local vertical winds (Hedenström and Alerstam, 1994; Nisbet, 1962; Piersma et al., 1997). Contrary to our predictions, and the assumption often made by optimal migration models (e.g. Alerstam, 1985; Pennycuick, 1969), the majority of descents were performed by active flight and the nightjars only sporadically descended by gliding or intermittent flight as recognised by the activity data. However, the median rate of descent during gliding was about a third of what was expected by a nightjar-shaped bird achieving its best glide, indicating that the gliding flights also included segments of flapping, outside the sampling period of the activity sensor. What motivates such behaviour is intriguing and these observations call for a re-consideration about the need to glide to efficiently allocate potential energy towards forward thrust (see below).

### Altitude variation within flights

Nightjars regularly changed flight altitude and the occurrence of long cruising phases with near-level flight, as generally assumed in migration models, was rather rare (e.g. Alerstam, 1985; Hedenström and Alerstam, 1994; Pennycuick, 1969). This work adds to a list of recent studies reporting surprisingly extensive vertical movements within migratory flights, hinting at a presumably widespread migration pattern that remains poorly understood (e.g. Bowlin et al., 2015; Liechti et al., 2018; Lindström et al., 2021; Senner et al., 2018; Sjöberg et al., 2018, 2021). Although near-level flight still made up a large fraction of the nightjars’ migratory flights (as revealed by the 5 min resolution data), it was commonly fragmented and intermixed with intermediate and large changes of altitude. The composition of these flight segments varied between seasons and regions as the nightjars spent a larger fraction of the time in level flight during autumn migration compared with spring, something also indicated by lower vertical mean speeds. These observations suggest that nightjars not only climb to higher altitudes during spring migration but also undertake more vertical movements during the ‘cruising’ phase of flights. In addition, the birds more frequently performed exploratory flights, resulting in flight termination during spring migration, probably because of the challenges of evaluating flight conditions at higher altitudes from the ground (Liechti, 2006; Schaub et al., 2004). Here, we extended the definition of exploratory flights to also include similar search-like vertical movements performed in the middle of a flight episode, resulting in similar seasonal distribution to that for the terminated exploratory flights. It is not clear what triggers birds to initiate such searches during their cruising flight, but given its seasonal difference it may be indicative of stable yet suboptimal (or a worsening of) flight conditions during spring, rather than a tactic of performing systematic weather updates across the whole air column. Radar observations have shown that avian migrants tend to concentrate at altitudes associated with local optima in flight conditions, even though still better conditions may occur at higher altitudes (Kemp et al., 2013). This supports the notion that birds could be able to sense windshear and adjust their flight altitude appropriately, but more rarely undertake vertical displacements to sample available tailwinds at a larger range of altitudes (Cochran and Kjos, 1985; Mateos-Rodríguez and Liechti, 2012).

### Cost of vertical displacements

A gain in flight altitude could be motivated by an adjustment towards improved wind conditions that reduce the overall cost of transport (COT), even though the ascent itself will increase the current flight costs. In ideal circumstances, however, the energy expenditure of a climb is conserved as potential energy that can be used to overcome drag during a gliding descent (Baudinette and Schmidt-Nielsen, 1974; Pennycuick, 1975). If the migrant uses this tactic, the added cost of a climb followed by a return to the starting altitude will be relatively small, although it is expected to vary to some degree with the bird's gliding performance and the metabolic cost to remain airborne when gliding. For the nightjars examined here, performing an exploratory movement at a climb rate of 1 m s^−1^ will increase COT by 7.9% relative to an uninterrupted level flight of the same horizontal distance (Fig. S3). Alternatively, the bird could remain in active flight during the descent. This scenario assumes that the potential energy gained contributes towards reducing the aerodynamic cost of forward flight while accounting for the bird's aerodynamic efficiency (Pennycuick, 1975). For the nightjars, this would increase COT by only 1.4% during the exploratory movement, assuming the vertical movement rates presented here (Fig. S3). The energy expenditure associated with vertical explorations will increase with the rates of ascent and descent but will still remain low for the range of vertical speeds operated by the nightjars. This suggests that descending by gliding flight is not a necessity for an efficient use of the potential energy and that migrants could remain in active flight regardless of their vertical movement. Like the nightjars tracked here, black-tailed godwits, *Limosa limosa*, also appear to continue flapping during descents (Senner et al., 2018). What our two scenarios have in common is that work during the climb is not wasted but invested in (and to a large degree recoverable from) the potential energy gained (Pennycuick, 1969). We therefore urge caution when using ‘climbing costs’ per se as an explanation for low-altitudinal flights if these costs are not well formulated, as it may be misleading and could obscure the effect of other undefined factors (Galtbalt et al., 2021). We hope our approach will inspire further analyses of the potential penalty of vertical movements and encourages researchers in attempting to enumerate such costs in future flight altitude-related studies.

### Conclusions

We present empirical data on the flight altitude selection of nightjars migrating between Europe and sub-Saharan Africa, including their dynamic use of near-level cruising flights and range of vertical displacement rates. We show that flight mechanical theory can provide a benchmark measure for the maximum rate of climb. However, our data also reveal that vertical displacements at rather low rates are performed throughout migratory flights, indicating regular adjustments of flight altitude while maintaining flapping flight. These seemingly effortless alterations of flight altitude by nightjars stand in sharp contrast to historical ideas of level cruising flights by avian migrants, but are in accordance with a scenario involving an efficient use of potential energy to power forward movement. To what extent these vertical movements influence the energy expenditure during flight among birds in general, and how migrants may balance potential additional costs against expected improvements of flight conditions are intriguing aspects for future studies.

## Supplementary Material

Supplementary information
