# Supplementary material for: Flight altitude dynamics of migrating European nightjars across regions and seasons
Source: J Exp Biol. 2021 Oct 25;224(20):jeb242836. doi: 10.1242/jeb.242836 (PMC8601712; doi:10.1242/jeb.242836)
Supplement: Supplementary information [file jexbio-224-242836-s1.pdf]

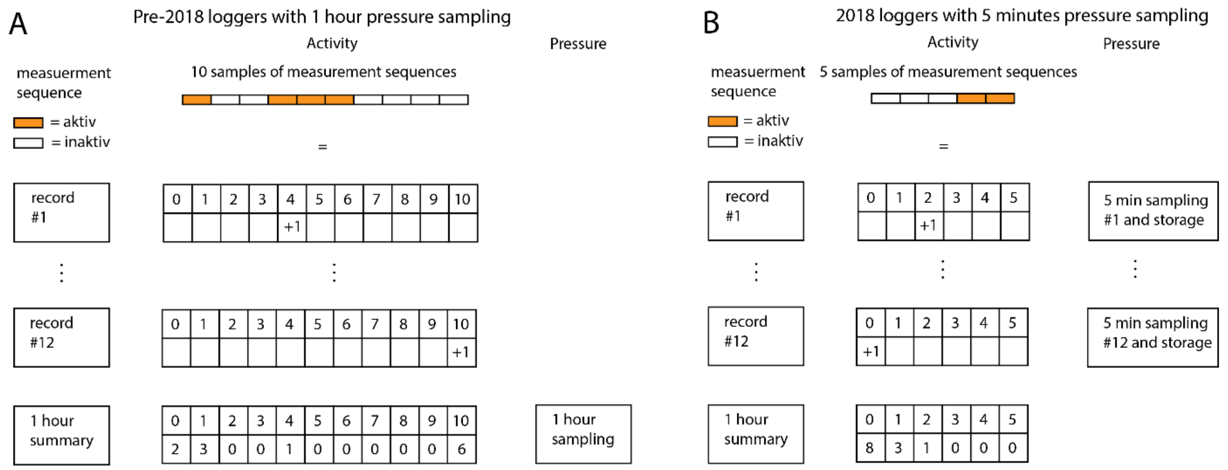

Fig. S1. Activity and altitude sampling of loggers deployed in 2016 and 2017 (A) and 2018 and 2019 (B). (A): Activity was sampled in a sequence of 10 measurements of 100 ms duration with 5 s between samples resulting in a record between 0 (no registered activity) and 10 (all samples indicated flight activity). The procedure was repeated every 5 minute and was summarized every hour when 12 records were collected. At every hour, a sample of the ambient pressure was sampled and stored.

Journal of Experimental Biology • Supplementary information

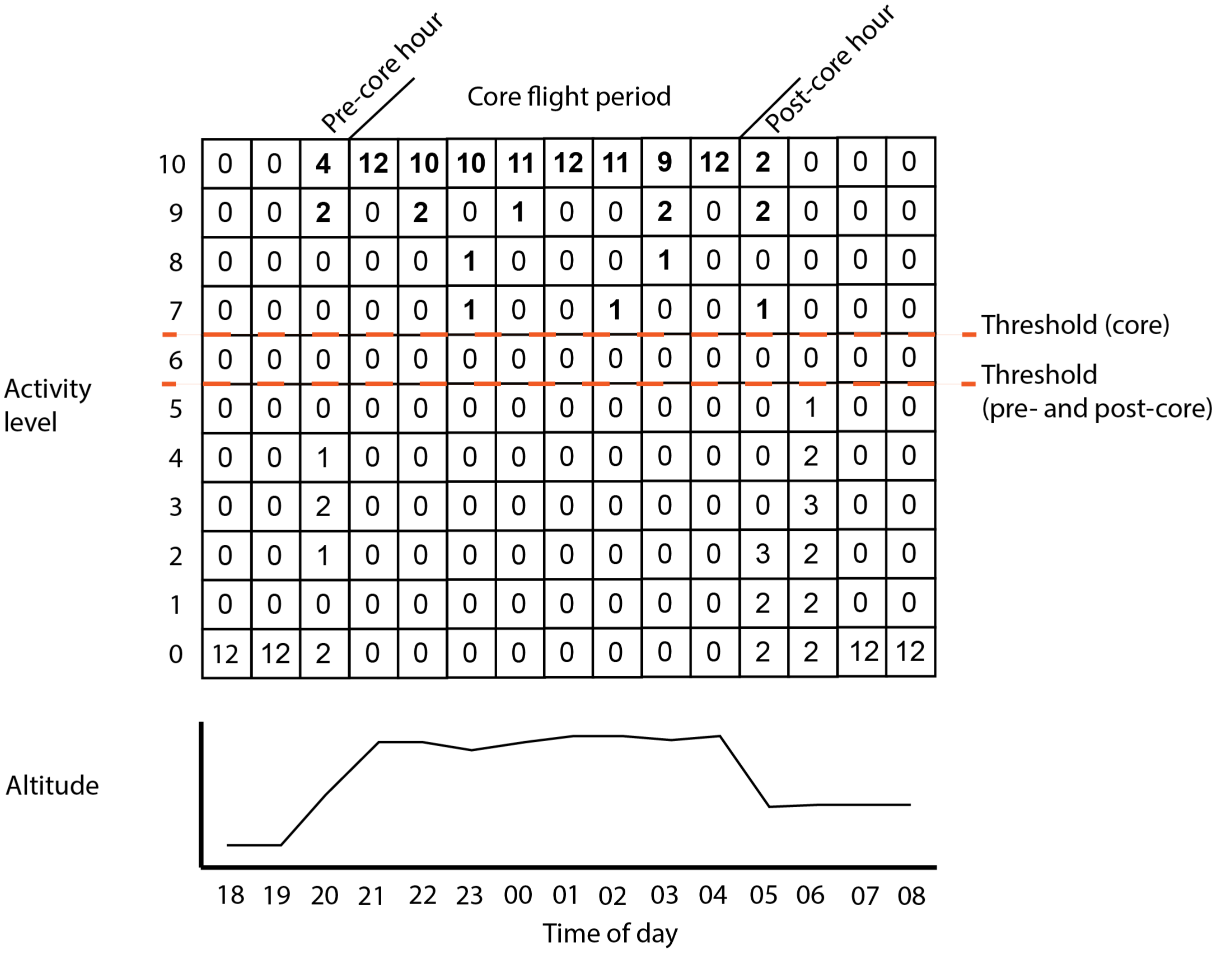

Fig. S2. Hourly activity summaries and altitude recording during a migration night as sampled by a pre-2018 logger. Every hour 12 activity records were stored based on the recorded activity level, from '0' = no activity, to '10' = always active (Fig. S1). Episodes of migratory flights were detected using an approach of two steps. First, the hourly number of activity records above a certain threshold were summarised (bold numbers). If the sum of activity records exceeded a predefined level, the hour was determined to belong to the core of the flight episode where the full hour was assumed to have been dedicated to the migratory flight. This usually resulted in several hours of continuous activity indicative for a migratory flight. Secondly, we inspected the hours immediately before and after the 'core' flight period in order to determine the number of 5-minutes registration of elevated activity presumably being a part of the 'core' flight episode (bold number). These were added to the flight episode to determine the timing and duration of flight episodes at a 5-minutes resolution. The recorded flight altitudes were used to visually confirm that the procedure detected episodes of migratory flights appropriately (Fig. 2).

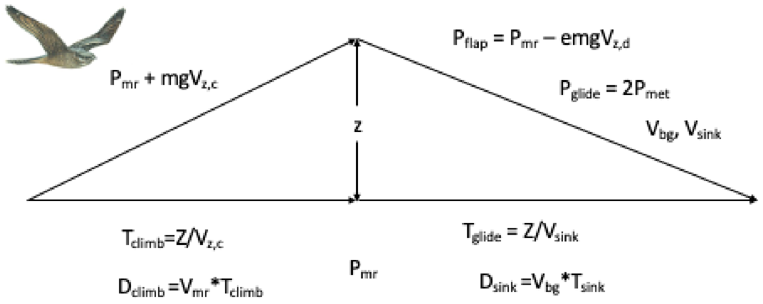

Fig. S3. A simple model to calculate the added cost for a bird to undertake a vertical exploratory movement relative to continuing a level cruising flight. Assume that a bird in level cruising flight wants to check the environmental (wind) conditions at a higher altitude than its current one, Z_0. First, as an example, we will consider a bird making a climb to a higher altitude during T_{climb} seconds at rate V_{climb} to reach an altitude $Z_0+T_{climb} \cdot V_{z,c}$, and thereby gain a potential energy mgZ (=mg $\cdot$ $T_{climb} \cdot V_{z,c}$). After a time T_{climb} the bird realizes the conditions are not better than at the original cruising altitude, and therefore decides to return to the original cruising altitude, Z_0. The descent to the original cruising altitude is assumed to be by gliding flight at a cost $2{\cdot}P_{met}$ (Baudinette and Schmidt-Nilesen 1974). We thereby assume that the potential energy gained during the climb is used to overcome the aerodynamic drag. To estimate realistic forward speed and sink rate during gliding flight for a common nightjar we calculated the glide polar (Pennycuick 1975), and assumed a common nightjar *Caprimulgus europaeus* could achieve almost a similar maximum lift to drag ratio (12.5) as a common swift *Apus apus* (Henningsson and Hedenström 2011), but since the common swift has a higher aspect ratio and the common nightjar has a higher body mass, we assume L:D = 11. Maximum range speed and associated power required to fly was calculated using the *afpt* package (Klein Heerenbrink et al. 2015). We compared the cost of transport (P/V = E/D) of conducting a vertical explorative deviation from the cruising altitude followed by a return to the original altitude with that of flying the same distance with powered flight at P_{mr}. For the assumptions given here and in Table S2, a climb at V_z = 1 m s^{-1} is associated with a 7.9% increase of cost of transport compared with an uninterrupted horizontal flight. The cost will increase with increasing metabolic cost of gliding flight and reduced glide performance (L:D). Second, if instead the descent phase is undertaken by flapping flight and assuming the potential energy gained contributes towards reducing the aerodynamic cost as Pflap = Pmr – emgVz,d, where e is an

Journal of Experimental Biology • Supplementary information

aerodynamic efficiency (cf. Pennycuick 1975). Again, given properties for a nightjar according to Table S2, an initial climb rate of $V_{z,c} = 0.67$ m s^{-1} and a descent rate of $V_{z,d} = 0.17$ m s^{-1} (as observed in this study), the cost of making a vertical exploratory flight and ten return to the original altitude is only 1.4% more than a horizontal flight the same distance. The cost will increase with both increasing climb rate and sink rate, but only to a small degree. The conclusion from these calculations is that the costs of vertical deviations from a constant cruising altitude is very low, simply because the potential energy gained can be used to alleviate the flight costs when descending. Notice that for these calculations we have disregarded the effects of changed air density with altitude and that the optimal cruising speed (V_{mr}) will be affected to a small degree when descending at reduced power. However, both these factors will have negligible effect on the cost estimates. Please note that here we only give an example calculation with the aim of illustrating the magnitude of what the cost can be of mid-flight explorative deviations. It has not escaped our notice that it is possible to extend this analysis to a more general situation, including scaling of body size and its impact on both powered and gliding flight performance.

Table S1. Sampling periods of available data recorded by the retrieved data loggers. Activity: the recording of activity by vertical acceleration; altitude: the sampling of ambient pressure; position: recorded periods of ambient light used for geolocation. Footnotes: (a): devices that have recorded activity, altitude and positioning data throughout the annual cycle according to the pre-set schedule; (b): devices that have recorded activity, altitude and positioning data but have stopped prematurely; (c): devices that have recorded activity, altitude and positioning accordingly, but where the pressure sensor has been blocked resulting in erroneous measurements.

Id	Activity		Altitude		Position	
	Start	Stop	Start	Stop	Start	Stop
$X500_b$	2016-07-14	2017-04-27	2016-07-14	2016-09-22	na	na
$X506_b$	2016-07-14	2017-05-22	2016-07-14	2016-12-03	na	na
X523	2016-07-15	2017-05-19	na	na	na	na
$X526_a$	2016-07-15	2017-05-19	2016-07-15	2017-05-19	2016-08-20	2016-08-24
					2016-10-20	2016-10-24
					2016-12-20	2016-12-24
					2017-02-20	2017-02-24
					2017-04-20	2017-04-24
X531	2016-07-15	2016-12-24	na	na	na	na
X539	na	na	na	na	na	na
X561	2016-07-15	2016-08-06	na	na	na	na
X572	2016-07-15	2017-05-25	na	na	2016-08-20	2016-08-24
					2016-10-20	2016-10-24
					2016-12-20	2016-12-24
					2017-02-20	2017-02-24
					2017-04-20	2017-04-24
X602	2016-07-15	2016-10-08	na	na	na	na
X627	2016-07-15	2017-06-18	na	na	2016-08-20	2016-08-24
					2016-10-20	2016-10-24
					2016-12-20	2016-12-24
					2017-02-20	2017-02-24
					2017-04-20	2017-04-24
$X630_b$	2016-07-15	2017-05-30	2016-07-15	2016-09-05	2016-08-20	2016-08-24
					2016-10-20	2016-10-24
					2016-12-20	2016-12-24
					2017-02-20	2017-02-24
					2017-04-20	2017-04-24
X787	2016-07-15	2017-03-28	na	na	2016-08-20	2016-08-24

Journal of Experimental Biology • Supplementary information

					2016-10-20	2016-10-24
					2016-12-20	2016-12-24
					2017-02-20	2017-02-24
					2017-04-20	2017-04-24
XD65	na	na	na	na	na	na
XD77	2017-08-01	2017-10-27	na	na	na	na
XD86$_a$	2017-08-01	2018-06-08	2017-08-01	2018-06-08	2017-08-06	2017-08-10
					2017-09-06	2017-09-10
					2017-10-06	2017-10-10
					2017-11-06	2017-10-10
					2017-12-06	2017-12-10
					2018-01-06	2018-01-10
					2018-02-06	2018-02-10
					2018-03-06	2018-03-10
					2018-04-06	2018-04-10
					2018-05-06	2018-05-10
					2018-06-06	2018-06-10
XD87	2017-08-10	2018-06-10	2017-08-10	2018-06-10	na	na
XF64$_a$	2018-08-01	2019-06-21	2018-08-01	2018-11-16	2018-08-06	2018-08-10
					2018-09-06	2018-09-10
					2018-10-06	2018-10-10
					2018-11-06	2018-11-10
					2018-12-06	2018-12-10
					2019-01-06	2019-01-10
			2019-01-18	2019-06-15	2019-02-06	2019-02-10
					2019-03-06	2019-03-10
					2019-04-06	2019-04-10
					2019-05-06	2019-05-10
					2019-06-06	2019-06-10
XF65$_a$	2018-08-01	2019-07-31	2018-08-01	2018-11-16	2018-08-06	2018-08-10
					2018-09-06	2018-09-10
					2018-10-06	2018-10-10
					2018-11-06	2018-11-10
					2018-12-06	2018-12-10
					2019-01-06	2019-01-10
			2019-01-18	2019-06-15	2019-02-06	2019-02-10
					2019-03-06	2019-03-10

Journal of Experimental Biology • Supplementary information

					2019-04-06	2019-04-10
					2019-05-06	2019-05-10
					2019-06-06	2019-06-10
					2019-07-06	2019-07-10
XF72$_a$	2018-08-01	2019-06-13	2018-08-01	2018-11-16	2018-08-06	2018-08-10
					2018-09-06	2018-09-10
					2018-10-06	2018-10-10
					2018-11-06	2018-11-10
					2018-12-06	2018-12-10
					2019-01-06	2019-01-10
			2019-01-18	2019-06-13	2019-02-06	2019-02-10
					2019-03-06	2019-03-10
					2019-04-06	2019-04-10
					2019-05-06	2019-05-10
					2019-06-06	2019-06-10
XF80$_c$	2018-08-01	2019-06-30	2018-08-01	2018-11-16	2018-08-06	2018-08-10
					2018-09-06	2018-09-10
					2018-10-06	2018-10-10
					2018-11-06	2018-11-10
					2018-12-06	2018-12-10
					2019-01-06	2019-01-10
			2019-01-18	2019-06-15	2019-02-06	2019-02-10
					2019-03-06	2019-03-10
					2019-04-06	2019-04-10
					2019-05-06	2019-05-10
					2019-06-06	2019-06-10
XF85$_a$	2018-08-01	2019-06-25	2018-08-01	2018-11-16	2018-08-06	2018-08-10
					2018-09-06	2018-09-10
					2018-10-06	2018-10-10
					2018-11-06	2018-11-10
					2018-12-06	2018-12-10
					2019-01-06	2019-01-10
			2019-01-18	2019-06-15	2019-02-06	2019-02-10
					2019-03-06	2019-03-10
					2019-04-06	2019-04-10
					2019-05-06	2019-05-10
X1F3$_a$	2019-08-01	2020-06-01	2019-08-01	2020-06-01	2019-08-20	2019-08-24

Journal of Experimental Biology • Supplementary information

					2019-09-20	2019-09-24
					2019-10-20	2019-10-24
					2019-11-20	2019-11-24
					2019-12-20	2019-12-24
					2020-01-20	2020-01-24
					2020-02-20	2020-02-24
					2020-03-20	2020-03-24
					2020-04-20	2020-04-24
					2020-05-20	2020-05-24
X1F4$_a$	2019-08-01	2020-06-23	2019-08-01	2020-06-23	2019-08-20	2019-08-24
					2019-09-20	2019-09-24
					2019-10-20	2019-10-24
					2019-11-20	2019-11-24
					2019-12-20	2019-12-24
					2020-01-20	2020-01-24
					2020-02-20	2020-02-24
					2020-03-20	2020-03-24
					2020-04-20	2020-04-24
					2020-05-20	2020-05-24
X1F5$_a$	2019-08-01	2020-06-27	2019-08-01	2020-06-27	2019-08-20	2019-08-24
					2019-09-20	2019-09-24
					2019-10-20	2019-10-24
					2019-11-20	2019-11-24
					2019-12-20	2019-12-24
					2020-01-20	2020-01-24
					2020-02-20	2020-02-24
					2020-03-20	2020-03-24
					2020-04-20	2020-04-24
X1FE$_a$	2019-08-01	2020-05-29	2019-08-01	2020-05-29	2019-08-20	2019-08-24
					2019-09-20	2019-09-24
					2019-10-20	2019-10-24
					2019-11-20	2019-11-24
					2019-12-20	2019-12-24
					2020-01-20	2020-01-24
					2020-02-20	2020-02-24
					2020-03-20	2020-03-24
					2020-04-20	2020-04-24

Journal of Experimental Biology • Supplementary information

$X20B_a$	2019-08-01	2020-06-03	2019-08-01	2020-06-03	2019-08-20	2019-08-24
					2019-09-20	2019-09-24
					2019-10-20	2019-10-24
					2019-11-20	2019-11-24
					2019-12-20	2019-12-24
					2020-01-20	2020-01-24
					2020-02-20	2020-02-24
					2020-03-20	2020-03-24
					2020-04-20	2020-04-24

Journal of Experimental Biology • Supplementary information

Table S2. Parameters and variables used in Fig. S3. Morphological properties represent a European nightjar (Tabel 1; *Caprimulgus europaeus;* Linnaeus).

Parameter/Variable	Definition
m	Body mass (0.07357 kg), this study
b	Wing span (0.5745 m), this study
S	Wing area (0.04239 m^2), this study
AR	Aspect ratio (7.8)
P_{mr}	Mechanical power required to fly at V_{mr} (0.698 W)
P_{met}	Basal metabolic rate (η 3.79 $m^{0.732}$) (W)
η	Energy conversion efficiency (0.23)
V_{mr}	Maximum range speed (8.65 m/s)
$V_{z,c}$ $V_{z,d}$	Climb/descent rate during flapping flight
V_{sink}	Sink rate during gliding descent (0.91 m/s)
V_g	Glide speed at maximum L:D (10 m/s)
$L{:}D_{max}$	11
k	Induced drag factor in gliding flight (1.1)
C_{dw}	Drag of the wings in gliding flight (0.014)
Z_0	Original cruising altitude
Z	Altitude reached during T_{climb}
T_{climb}	Duration of ascent
T_{decent}	Duration of descent back to Z_0 given $V_{z\ d}$
T_{sink}	Time of gliding flight from Z to Z_0
D_{climb}	Horizontal displacement during the climb
D_{glide}	Horizontal displacement during the glide from Z to Z_0
g	Acceleration due to gravity (9.81 m s^{-2})
e	Aerodynamic efficiency (0.9)

References

Baudinette, R. V. and Schmidt-Nilesen, K. (1974). Energy cost of gliding flight in herring gulls. *Nature* **248**, 83-84.

Henningsson, P. and Hedenström, A. (2011). Aerodynamics of gliding flight in common swifts. *J. Exp. Biol.* **214**, 382-393.

Klein Heerenbrink, M., Johansson, L. C. and Hedenström, A. (2015). Power of the wingbeat: modelling the effects of flapping wings in vertebrate flight. *Proc. R. Soc. A* **471**, 20140952

Pennycuick, C. J. (1975). Mechanic of flight In *Avian Biology* (ed. D. S. Farner, J. R. King, and K. C. Parkes), Vol. **5**, 1-75. Academic press, New York.

Journal of Experimental Biology • Supplementary information
